# Alterations of Brain Structural and Functional Connectivity Networks and Its Correlations With Cognitive Function in Patients With Hypothalamic Syndrome Following Craniopharyngioma Resection

**DOI:** 10.1002/brb3.70730

**Published:** 2025-08-27

**Authors:** Shuang Li, Junya Ma, Zanyong Tong, Hongting Jiang, Lusheng Li, Yuting Zhang

**Affiliations:** ^1^ Department of Radiology Children's Hospital of Chongqing Medical University; National Clinical Research Center For Child Health and Disorders; Ministry of Education Key Laboratory of Child Development and Disorders; China International Science and Technology Cooperation Base of Child Development and Critical Disorders; Chongqing Key Laboratory of Child Neurodevelopment and Cognitive Disorders Chongqing China; ^2^ Department of Neurosurgery Children's Hospital of Chongqing Medical University; National Clinical Research Center For Child Health and Disorders; Ministry of Education Key Laboratory of Child Development and Disorders; China International Science and Technology Cooperation Base of Child Development and Critical Disorders; Chongqing Key Laboratory of Child Neurodevelopment and Cognitive Disorders Chongqing China

**Keywords:** Craniopharyngioma, functional connectivity networks, graph theory, hypothalamic syndrome, structural connectivity networks

## Abstract

**Objective:**

This study aims to investigate the alterations in structural and functional connectivity networks (SCN and FCN) in children with hypothalamic syndrome (HS) following craniopharyngioma resection and to explore the relationship between these network changes and clinical manifestations.

**Materials and Methods:**

We performed graph theory analysis on SCN and FCN derived from 36 patients with HS and 36 age‐ and sex‐matched healthy controls (HC), with an age range of 6 to 13 years. We evaluated characteristics, nodal properties, and the coupling between SCN and FCN across 90 brain nodes. Partial correlation analyses examined relationships between graph theory properties and clinical scales, including the Wechsler Intelligence Scale for Children (WISC), the Wechsler Memory Scale (WMS), and the Attention Deficit Hyperactivity Disorder (ADHD) scale.

**Results:**

The SCN in the HS group exhibited abnormal global properties, including increased characteristic path length (Lp), decreased global efficiency (Eg), and local efficiency (ELOC), alongside notable reductions in nodal properties, such as degree centrality (Dc) and nodal efficiency (Ne) across multiple nodes. The FCN in the HS group also displayed abnormal global attributes, with elevated Lp and reduced Eg, alongside decreased Dc at the median cingulate and paracingulate gyri (DCG.L) node. However, no statistically significant differences were found in structural‐functional connectivity (SC‐FC) coupling between groups. Correlation analysis revealed significant links between WISC, WMS, and ADHD scales and various graph‐theoretic properties in the HS group.

**Conclusion:**

In patients with HS following craniopharyngioma resection, alterations in SCN and FCN characteristics have been observed. These neural changes are associated with cognitive developmental impairments related to HS, providing neuroimaging evidence elucidating the mechanisms underlying cognitive deficits in HS patients.

## Graphical abstract

After preprocessing the rs‐fMRI and DTI data, nodes of the functional connectivity network (FCN) and structural connectivity network (SCN) were defined based on the Automated Anatomical Labeling (AAL 90) atlas consisting of 90 regions. Subsequently, the FCN and SCN were constructed accordingly. Using graph theory analysis methods, relevant global and nodal topological metrics were calculated for both networks. Statistical analyses were performed to identify global and nodal metrics exhibiting significant differences. These significantly different metrics were then subjected to partial correlation analysis with corresponding cognitive developmental deficit scale scores to obtain the relevant partial correlation results.

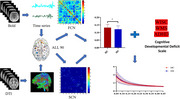

## 1 Introduction

The hypothalamus, located at the base of the diencephalon, is essential for regulating neuroendocrine activity (Burbridge et al. 2016). Lesions or injuries to this area can lead to hypothalamic syndrome (HS), characterized by various clinical symptoms (Cemeroglu et al. 2015, Müller et al. 2022). Common causes of HS include tumors in the sella turcica, such as craniopharyngiomas, germ cell tumors, gliomas, and Rathke's cleft cysts, as well as genetic disorders like Prader‐Willi syndrome and septo‐optic dysplasia (Müller et al. 2022, van Santen et al. 2023). Craniopharyngiomas are particularly frequent, with treatment typically focused on complete surgical resection (Li et al. 2024). Clinical manifestations of HS include persistent weight gain, endocrine dysfunction, memory issues, attention deficits, impaired impulse control, and an increased risk of cardiovascular and metabolic disorders (Müller, 2019, Müller 2018). In children, symptoms may also encompass obesity, pituitary dysfunction, diabetes insipidus, temperature instability, and sleep disorders. Behavioral problems, such as aggression and reduced impulse control, are common, especially in patients with sellar tumors undergoing surgery (Müller et al. 2022, Pascual et al. 2018). These developmental, psychiatric, and behavioral manifestations inevitably lead to both structural and functional alterations in the brains of HS patients. Diagnosis is guided by criteria established by Hermann L. Müller and colleagues, aligning with current standards (Müller 2017, Müller et al. 2011, Elliott et al. 2010). Research on brain development in individuals with HS is limited due to its varied etiologies.

Brain networks demonstrate complex connections, balancing global efficiency with local specialization (Ding et al. 2024). Functional connectivity networks (FCN) from resting‐state functional magnetic resonance imaging (rs‐fMRI) and structural connectivity networks (SCN) from diffusion tensor imaging (DTI) are currently among the most widely used methodologies in brain network research (Abdolalizadeh et al. 2023, Bullmore and Sporns 2009). Previous studies have examined the functional connectivity of the hypothalamus during resting states, revealing that the hypothalamus is primarily connected to the striatum, midbrain, thalamus, insula, frontal lobe, cingulate cortex, temporal cortex, and parts of the cerebellum, with robust interactions with these areas (Kullmann and Veit 2021). Furthermore, the study by Huang Z and colleagues demonstrated alterations in the frontal motivational circuits and limbic system in Prader‐Willi syndrome, correlating these changes with genetic and clinical outcomes (Huang and Cai 2023). Additionally, children with Prader‐Willi syndrome exhibited (1) reduced structural‐functional coupling, (2) increased characteristic path length and decreased global efficiency in structural networks at the global level, and (3) alterations in nodal properties of canonical cortical and subcortical networks characterized predominantly by reductions in structural networks and increases in functional networks (Huang et al. 2024). However, most investigations have focused on anomalies within the FCN and SCN, often overlooking their intricate interactions. Utilizing structural‐functional connectivity (SC‐FC) coupling analysis can enhance sensitivity to disruptions in brain networks across multiple neuroimaging modalities, providing a more comprehensive understanding (Honey et al. 2009, Kong et al. 2021).

Currently, there is limited understanding of brain network patterns in children with HS and the relationships between SCN and FCN. Our study aimed to investigate (1) the alterations in the topological organization of SCN and FCN networks in pediatric HS patients following craniopharyngioma resection using graph theory analysis, as well as the relationship between functional and structural coupling, and (2) the associations between brain network changes and developmental status related to cognitive dysfunction in HS, assessed via partial correlation analysis.

## 2 Materials and Methods

### 2.1 Participants

In accordance with the ethical principles of the Declaration of Helsinki, written informed consent was obtained from all participants' parents or legal guardians. Additionally, for adolescent participants aged ≥ 12 years, individual assent was secured to ensure their voluntary participation. This study received ethical approval from the Ethics Committee of the Children's Hospital of Chongqing Medical University: Approval Number is File No. (2023) Review Board (Research) No. 601.

From May 2023 to August 2024, we prospectively recruited 36 patients aged 6 to 13 years who were diagnosed with HS following complete resection of craniopharyngioma, along with age‐ and sex‐matched normally developing children as healthy controls (HC). The HS group comprised 24 males and 12 females, with a mean age of 8.91 ± 2.29 years, while the HC group included 20 males and 16 females, with a mean age of 9.18 ± 2.01 years. The HS diagnosis followed international consensus standards established by Hermann L. Müller and colleagues, consistent with the latest criteria proposed by Hanneke M. van Santen and colleagues (Müller et al. 2022, van Santen et al. 2023). All participants were not on sedative medications during assessments, and those unable to cooperate or sign consent were excluded.

Inclusion criteria for the HS group: (1). Age between 6 and 16 years; (2). Right‐handedness; (3). Follow‐up at least one month after complete resection of craniopharyngioma; (4). Diagnosis of HS confirmed by two qualified pediatric neurosurgeons through clinical observation, adhering to the international diagnostic standards and consensus established by Hermann L. Müller and colleagues. Inclusion criteria for the HC group: (1). Age between 6 and 13 years; (2). Right‐handedness; (3). Normal developing children with no history of neurological disorders and no abnormal findings on routine magnetic resonance imaging (MRI).

Exclusion criteria for both HS and HC groups: (1). Head motion exceeding 2 mm or rotation greater than 2° during the MRI scanning process; (2). Any contraindications for MRI.

Clinical Assessment: Children with HS had no tumor recurrence prior to enrollment and were managed according to the “National United Kingdom guidelines for the management of pediatric craniopharyngioma” (Gan et al. 2023). Following MRI scanning, clinical information for both groups of children was recorded immediately.

Additionally, the HS group underwent assessments that included the duration of the disease, clinical manifestations, the Wechsler Intelligence Scale for Children (WISC), the Wechsler Memory Scale (WMS), and the Attention Deficit Hyperactivity Disorder (ADHD) scale.

### 2.2 Imaging Acquisition

During the MRI scanning procedure, all participants were instructed to remain calm, and portable metallic and electronic items, as well as any related contraindications for the scan, were removed in advance. Furthermore, all subjects were required to wear soft earplugs and utilize soft foam padding to minimize head movement, remaining awake and still throughout the entire scanning process. High‐resolution 3D T1‐weighted imaging (3D T1WI), rs‐fMRI, and DTI data were sequentially acquired using a 3.0 Tesla MRI scanner (Discovery MR750, GE Healthcare, Milwaukee, WI, United States) equipped with an eight‐channel phased‐array head coil. The scanning parameters are provided in Supplementary Text S1 within the Supplementary Materials.

Quality assurance checks were conducted on all 3D T1WI, rs‐fMRI, and DTI data to exclude scans with excessive motion and/or artifacts following preprocessing corrections. There were no statistically significant differences in head motion parameters between groups, with maximum movement and angular rotation both less than 2°. A total of six subjects were deemed ineligible during the preprocessing phase (four from the HS group and two from the HC group) and were excluded. Analysis was subsequently performed on the remaining 36 subjects from the HS group and 36 subjects from the HC group.

### 2.3 Data Preprocessing

The preprocessing and analysis of rs‐fMRI and 3D T1WI data were conducted using the Sales Process Management 12 (SPM12; https://www.fil.ion.ucl.ac.uk/spm/) software package on the MATLAB platform (version 2013b; www.csie.ntu.edu.tw~cjlin/libsvm) and the graph theory analysis GRETNA toolbox (version 2.0.0; https://www.nitrc.org/projects/gretna/).

The preprocessing of the rs‐fMRI data included the following steps: (1) Removal of Initial Time Points: the first 10 time points were excluded to allow the MRI signal to reach a stable state; (2) Motion Correction: temporal and head motion correction was performed on the remaining images (participants exhibiting head motion exceeding 2 mm in the x, y, or z axes, or any angular rotation exceeding 2°, were excluded from analysis); (3) Normalization: structural images (3D T1WI) were normalized to the Montreal Neurological Institute (MNI) space and resampled to achieve a voxel size of 3 × 3 × 3 mm^3^; (4). Linear Trend Removal: the linear trends of the blood oxygen level‐dependent (BOLD) signal time course were removed; (5) Noise Denoising: noise removal included regressing out Friston‐24 head motion parameters, cerebrospinal fluid signals, and white matter signals; (6) Low‐Frequency Filtering: a band‐pass filter (0.01 ≤ f ≤ 0.08 Hz) was applied to eliminate the effects of high‐frequency physiological noise (Tan et al. 2019, Liao et al. 2024).

For the preprocessing and network matrix construction of DTI data, the PANDA software (version 1.3.1; https://www.nitrc.org/projects/panda/) based on FSL (version 5.0.9; https://fsl.fmrib.ox.ac.uk/fsl/fslwiki/) was utilized (Cocchi et al. 2014). The main processing steps for DTI data included the following steps: (1) Format Conversion: conversion of DTI raw DICOM format data into 4D NIFTI format; (2) Resampling: resampling of the data for improved consistency and accuracy in analysis; (3) Motion and Eddy Current Correction: correction of head motion and eddy currents; (4) Skull Stripping: removal of non‐brain tissues and skull from the imaging data; (5) Spatial Registration: alignment of images to standard space; (6). White Matter Fiber Reconstruction: Whole‐brain white matter fiber reconstruction was performed using the deterministic fiber tracking algorithm known as Fiber Assignment by Continuous Tracking (FACT), which averaged across multiple directions and calculated diffusion tensor indices. Deterministic fiber tracking was terminated under the following criteria: fractional anisotropy (FA) < 0.2 or tracking angle > 45°.

### 2.4 Brain Connectivity Network Construction

**FIGURE 1:**
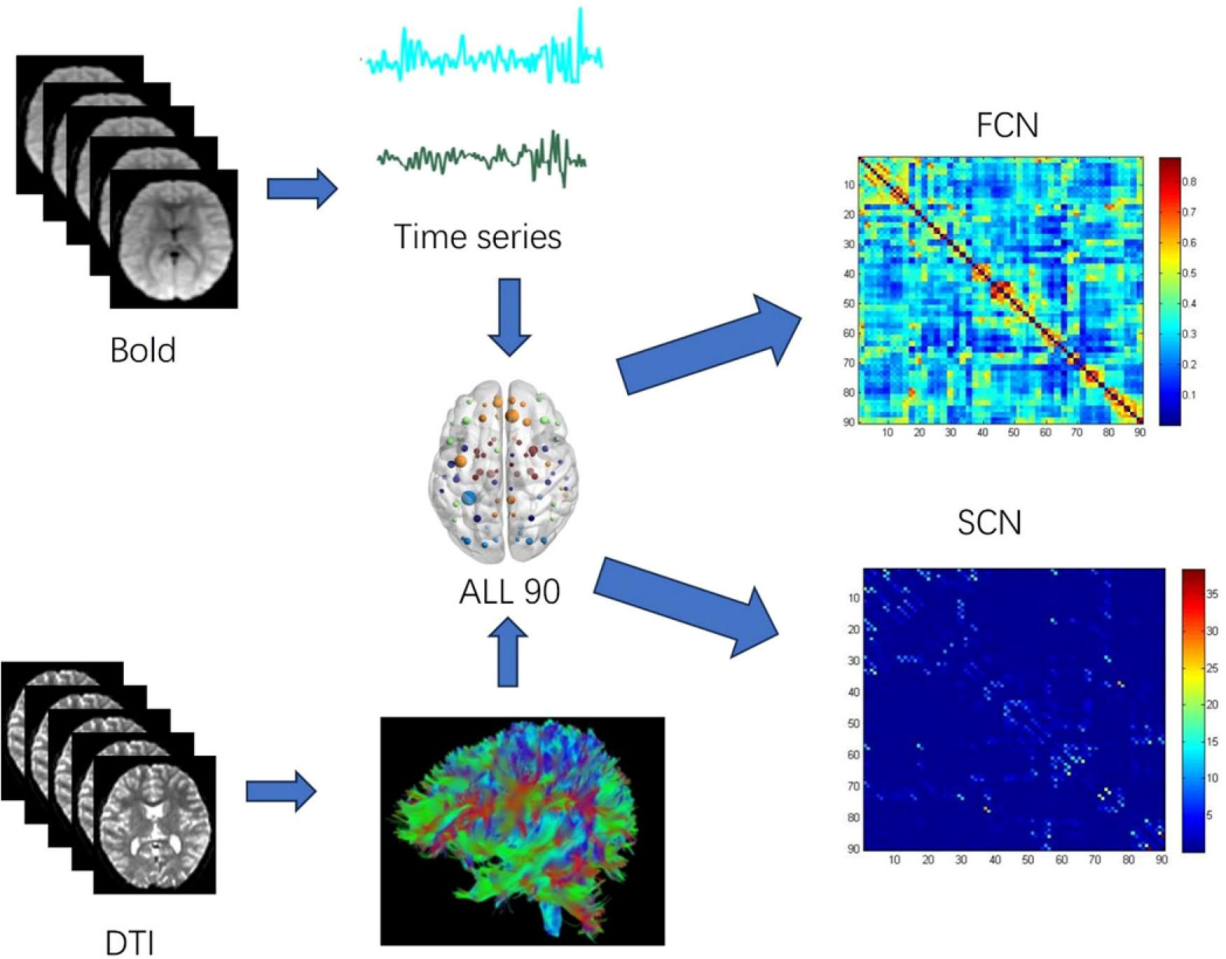
Flowchart of brain network construction. After preprocessing the time series of rs‐fMRI and performing deterministic fiber tracking using DTI, nodes (derived from the AAL atlas) and weighted edges were defined to obtain the FCN and SCN for each participant, respectively.

The FCN and SCN were derived from the preprocessed rs‐fMRI and DTI data, as illustrated in Figure 1. In this study, the nodes of both the FCN and SCN were defined based on the 90 regions of the Automated Anatomical Labeling (AAL90) atlas. For the FCN, the edge weights were calculated based on the average time series of the rs‐fMRI data between each pair of the 90 nodes, resulting in inter‐regional correlation coefficients. This quantifies the strength of functional connectivity between the regions. In the case of the SCN, the edge weights were defined by the product of the fractional anisotropy (FA) matrix and the fiber number (FN) matrix. This approach allowed us to establish a weighted connectivity matrix for the 90 nodes, reflecting the structural connections based on the integrity and quantity of the white matter fibers connecting the regions (Xu et al. 2022).

### 2.5 Graph Theory Analysis

The graph theory properties of the SCN and FCN were calculated using the GRETNA 2.0 software package based on MATLAB (https://www.nitrc). The sparse thresholds for FCN and SCN ranged from 0.05 to 0.45, with a step size of 0.01.

Global network characteristics of both the FCN and SCN were represented through various metrics, including the clustering coefficient (Cp), characteristic path length (Lp), global efficiency (Eg), local efficiency (Eloc), normalized clustering coefficient (γ), normalized characteristic path length (λ), and small‐worldness (σ). For the calculation of small‐world attributes, a random network sample size of 1000 was selected. Regional network characteristics were delineated using degree centrality (Dc), betweenness centrality (Bc), node efficiency (Ne), node local efficiency (Nle), and node clustering coefficient (NCp).

Notably, the area under the curve (AUC) was employed to summarize the topological descriptions of the SCN and FCN across different sparsity thresholds. This approach effectively captures alterations in brain networks associated with neurological diseases without relying on any single threshold selection (Wang et al. 2019).

### 2.6 Structural‐Functional Connectivity Coupling Analysis

In this study, non‐zero structural connections from the connectivity matrix of each subject were selected and re‐scaled to a Gaussian distribution. The functional connectivity network was then extracted from these selected structural connections. Pearson correlation analysis was performed between the functional and structural connectivity values, resulting in a single SC‐FC coupling value for each participant (Kim et al. 2019, Honey et al. 2009).

### 2.7 Statistical Analysis

Statistical analyses were performed by using Statistical Product and Service Solutions version 26.0 (SPSS, IBM Corp., NY, United States). The Kolmogorov–Smirnov test was performed on demographic and laboratory data to test the normality of continuous variables. Intergroup differences in normally distributed and non‐normally distributed continuous data were assessed by using two‐tailed independent samples *t‐*tests and Mann–Whitney *U* tests, respectively. Intergroup differences in the categorical data (such as sex) were examined by using the χ‐squared test. The results are reported at the significance level of *p* < 0.05.

Intergroup differences in structural‐functional coupling, global, and nodal properties were analyzed by using general linear models on graph theoretical measures controlling for age, sex, and duration of illness. A significance level of *p* < 0.05 was used for global properties, while node properties were adjusted for multiple comparisons with a Bonferroni correction (*p* < 0.0005556) (Zhang et al. 2019). SC‐FC coupling values between HS and HC groups were compared using 5000 permutations.

Additionally, partial correlation analyses explored correlations between graph theory properties in the HS group and clinical scale scores, controlling for age, sex, and duration of illness, setting a threshold at *p* < 0.05 (Zhuo et al. 2021).

## 3 Result

### 3.1 Demographic and Clinical Characteristics

After MRI preprocessing and quality control, 36 participants from the HS group and 36 from the HC group were included in the statistical analyses. Table 1 summarizes demographic and clinical characteristics, showing no statistically significant differences in age or sex between the groups (both *p* > 0.05).

**TABLE 1:** Demographic and clinical data.

Characteristic	HS	HC	*p* value
Patients (*n*)	36	36	
Age (years, avg)	8.91 ± 2.29	9.18 ± 2.01	0.587 ^a^
Gender (Males/Females, *n*)	24/12	20/16	0.334 ^b^
Disease Duration (months, avg)	4.89 ± 3.11	N/A	
Clinic grading (*n*)			
IGrade	16	N/A	
IIGrade	7	N/A	
III Grade	13	N/A	—
Clinic scales			
WISC (avg)	73.64 ± 12.36	N/A	
WMS (avg)	75.08 ± 10.95	N/A	
ADHD (avg, %)	24.61 ± 8.15	N/A	

*Note*: a for *t*‐test, b for χ2‐test. The significance level was set at *p* < 0.05.

**Abbreviations**: ADHD, Attention Deficit Hyperactivity Disorder scale; Clinic grading, Clinical Severity Grading of Hypothalamic Dysfunction; HC, healthy control; HS, hypothalamic syndrome; WISC, Wechsler Intelligence Scale for Children; WMS, Wechsler Memory Scale for Children.

### 3.2 Group Differences in Global Properties

**FIGURE 2:**
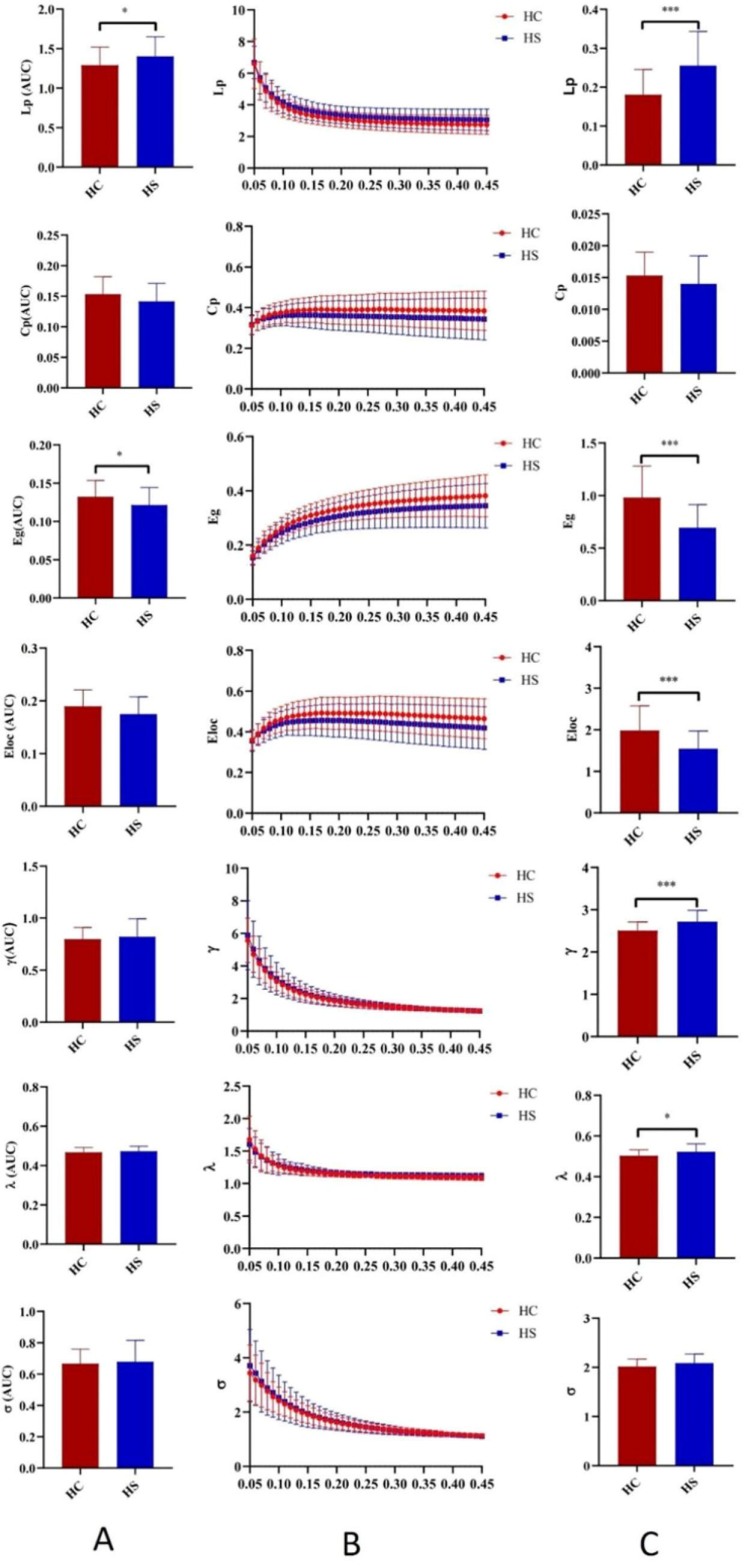
Global properties of functional and structural connectivity networks. For HC (red) and HS (blue), a series of sparsity thresholds (B) correspond to the global properties of the FCN and SCN as indicated in elements A and C. B illustrates the results derived from the range of sparsity values for the FCN, while columns A and C display the area under the curve results for the corresponding global properties of the FCN and SCN, respectively. *p for a significant statistical threshold of global properties was set at *p* < 0.05.

Both the HS and HC groups demonstrated small‐world organization in FCN and SCN (all γ > 1, λ ≈ 1, and σ > 1; Table 2). In the SCN, the HS group exhibited statistically significant increases in Lp, γ, and λ compared to the HC group, whereas Eg and Eloc were decreased (*p* < 0.05; Table 2 and Figure 2). For the FCN, the HS group exhibited an increase in Lp and a statistically significant decrease in Eg when compared to the HC group (*p* < 0.05; Table 2 and Figure 2).

**TABLE 2:** Global graph theoretical properties and coupling of the two groups.

Metrics	HC (Mean ± SD)	HS (Mean ± SD)	*p*
FCN			
Lp	1.2909 ± 0.2287	1.4038 ± 0.2453	0.047*
Cp	0.1533 ± 0.0286	0.1415 ± 0.0294	0.087
Eg	0.1322 ± 0.0212	0.1215 ± 0.0229	0.043*
Eloc	0.1896 ± 0.0315	0.1751 ± 0.0328	0.059
γ	0.7980 ± 0.1122	0.8209 ± 0.1735	0.507
λ	0.4676 ± 0.0234	0.4725 ± 0.0256	0.402
σ	0.6665 ± 0.0931	0.6785 ± 0.1374	0.665
SCN			
Lp	0.1806 ± 0.0648	0.2553 ± 0.0878	< 0.001*
Cp	0.0153 ± 0.0037	0.0140 ± 0.0044	0.167
Eg	0.9812 ± 0.3003	0.6950 ± 0.2183	< 0.001*
Eloc	1.9840 ± 0.5925	1.5465 ± 0.4238	0.001*
γ	2.5072 ± 0.2043	2.7169 ± 0.2668	< 0.001*
λ	0.5027 ± 0.0295	0.5222 ± 0.0395	0.020*
σ	2.0150 ± 0.1532	2.0877 ± 0.1837	0.106
Coupling	0.1107 ± 0.0720	0.1206 ± 0.0567	0.518

*Note*: *The significance threshold for global properties is set at *p* < 0.05.

**Abbreviations**: CP, clustering coefficient; Eg, global efficiency; Eloc, local efficiency; γ, normalized clustering coefficient; HC, healthy controls; HS, hypothalamic syndrome; λ, normalized characteristic path length; LP, characteristic path length; σ, small‐world.

### 3.3 Group Differences in Node Properties

**FIGURE 3:**
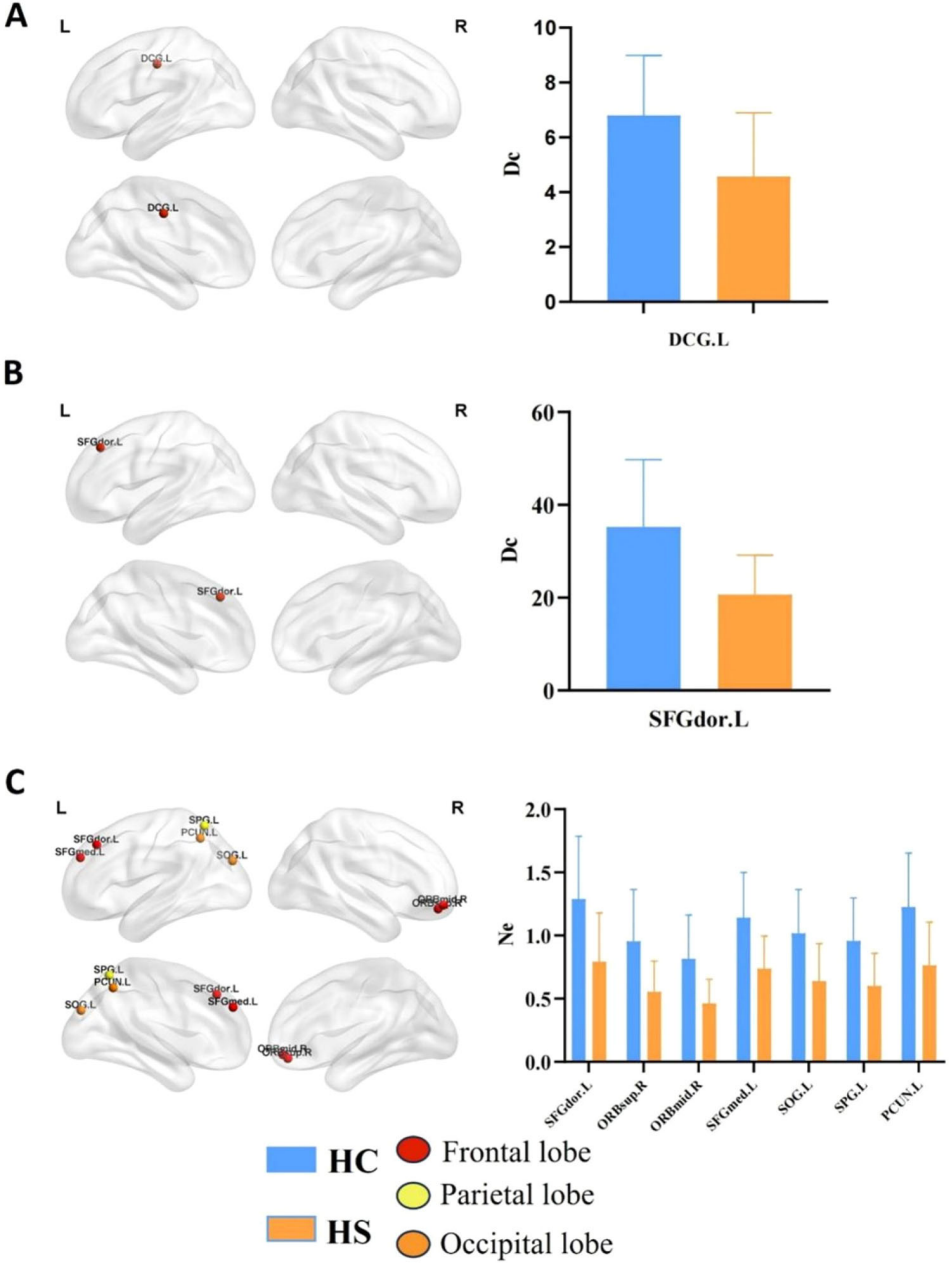
Changes in node attributes of functional and structural connectivity networks. Panel (A) shows the brain regions with significantly altered node characteristics in the Dc of FCN. Panels (B) and (C) depict the brain regions with significant changes in node characteristics for Dc and Ne in the SCN, respectively. The statistical significance threshold for node characteristics is set at *p* < 0.0005556 (0.05/90). Abbreviations for nodes can be found in the supplementary materials.

Regarding the FCN, comparison with the HC group revealed that only one node in the HS group exhibited a significant reduction in node properties: the median cingulate and paracingulate gyri (DCG.L), which showed a statistically significant decrease in Dc (*p* < 0.00001, Bonferroni correction; Table 3 and Figure 3A).

**TABLE 3:** Nodal graph theoretical properties of the two groups.

Properties	Networks	Nodes	HC (Mean ± SD)	HS (Mean ± SD)	*p* value
Dc	FCN	DCG.L	6.8058 ± 2.1797	4.5722 ± 2.3263	0.00008*
Dc	SCN	SFGdor.L	35.2440 ± 14.4928	20.6501 ± 8.5250	< 0.00001*
Ne	SCN	SFGdor.L	1.4100 ± 0.4770	0.8950 ± 0.2744	< 0.00001*
		ORBsup.R	0.9537 ± 0.4106	0.5535 ± 0.2443	< 0.00001*
		ORBmid.R	0.8153 ± 0.3466	0.4622 ± 0.1913	< 0.00001*
		SFGmed.L	1.1420 ± 0.3580	0.7379 ± 0.2573	< 0.00001*
		SOG.L	1.0171 ± 0.3475	0.6389 ± 0.2972	< 0.00001*
		SPG.L	0.9575 ± 0.3406	0.5784 ± 0.2728	< 0.00001*
		PCUN.L	1.2260 ± 0.4262	0.7636 ± 0.3428	< 0.00001*

*Note*: *The significance threshold for node attributes is set at *p* < 0.0005556 (0.05/90).

**Abbreviations**: DC, degree centrality; FCN, functional connectivity network; HC, healthy controls; HS, hypothalamic syndrome; NCp, node clustering coefficient; NE, node efficiency; NLe, local efficiency; SCN, structural connectivity network.

Abbreviations for nodes can be found in the supplementary materials.

In the SCN, the HS group exhibited a statistically significant decrease in Dc of the Superior frontal gyrus, dorsolateral (SFGdor.L), compared to the HC group (*p* < 0.00001, Bonferroni correction; Table 3 and Figure 3B). Additionally, a decreased Ne was observed in seven nodes of the HS group, including SFGdor.L, Superior frontal gyrus, orbital part (ORBsup.R), Middle frontal gyrus, orbital part (ORBmid.R), Superior frontal gyrus, medial (SFGmed.L), Superior occipital gyrus (SOG.L), Superior parietal gyrus (SPG.L), and Precuneus (PCUN.L) (*p* < 0.00001, Bonferroni correction; Table 3 and Figure 3C). Abbreviations for nodes are provided in the Supplementary Text S2.

### 3.4 SC‐FC Coupling Analysis Results

**FIGURE 4:**
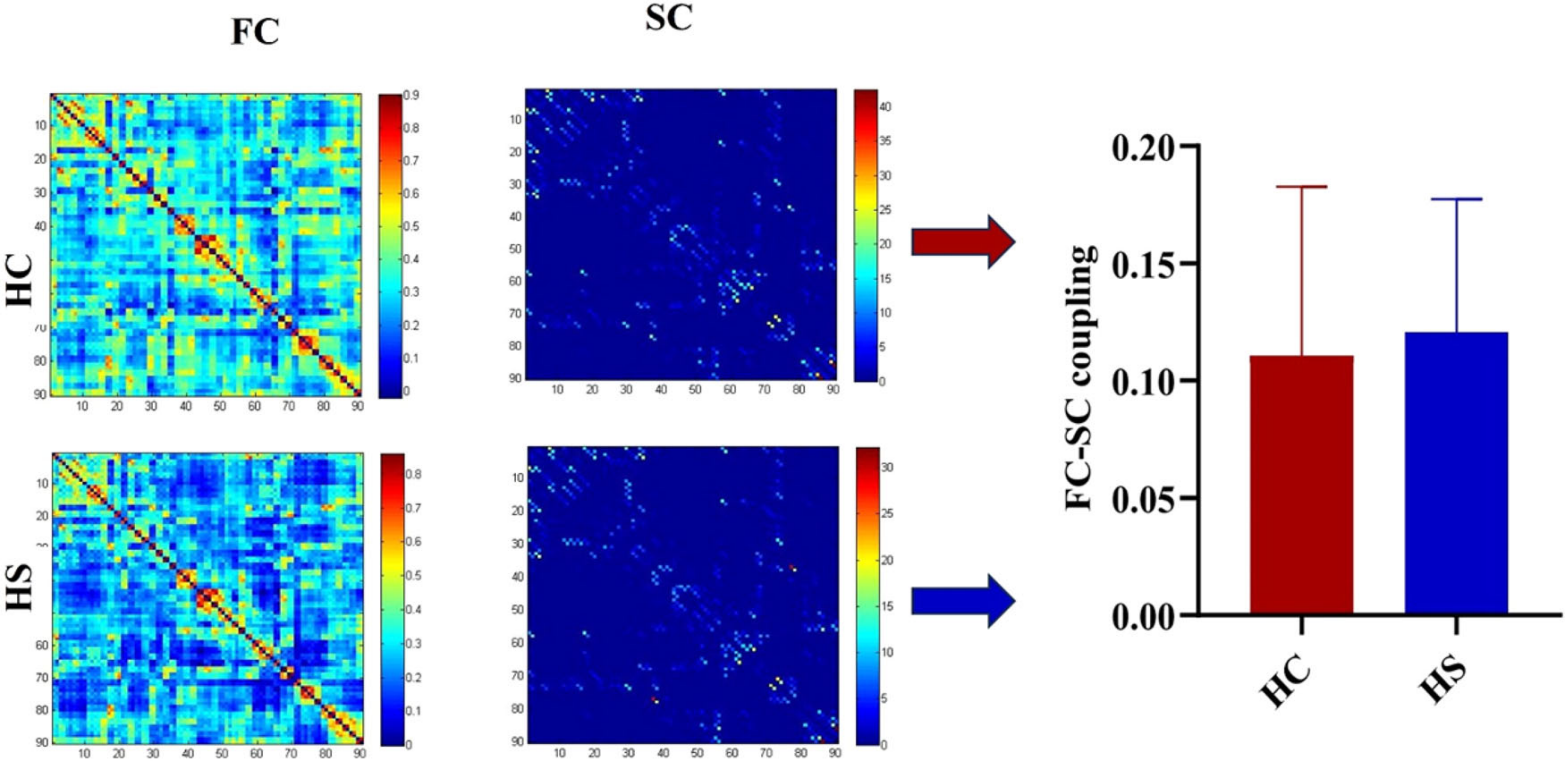
Structural‐Functional connectivity coupling of the two groups. The average SC‐FC coupling of the HC and HS groups. The results indicate that there is no significant difference in SC‐FC coupling between the two groups (*p* = 0.518, based on 5000 random permutation tests).

The examination of network structure‐functional coupling differences based on connection levels revealed no statistically significant differences between the HS and HC groups in terms of SC‐FC coupling (*p* > 0.05, 5000 permutations; Table 2 and Figure 4).

### 3.5 Partial Correlation Analysis Results

**FIGURE 5:**
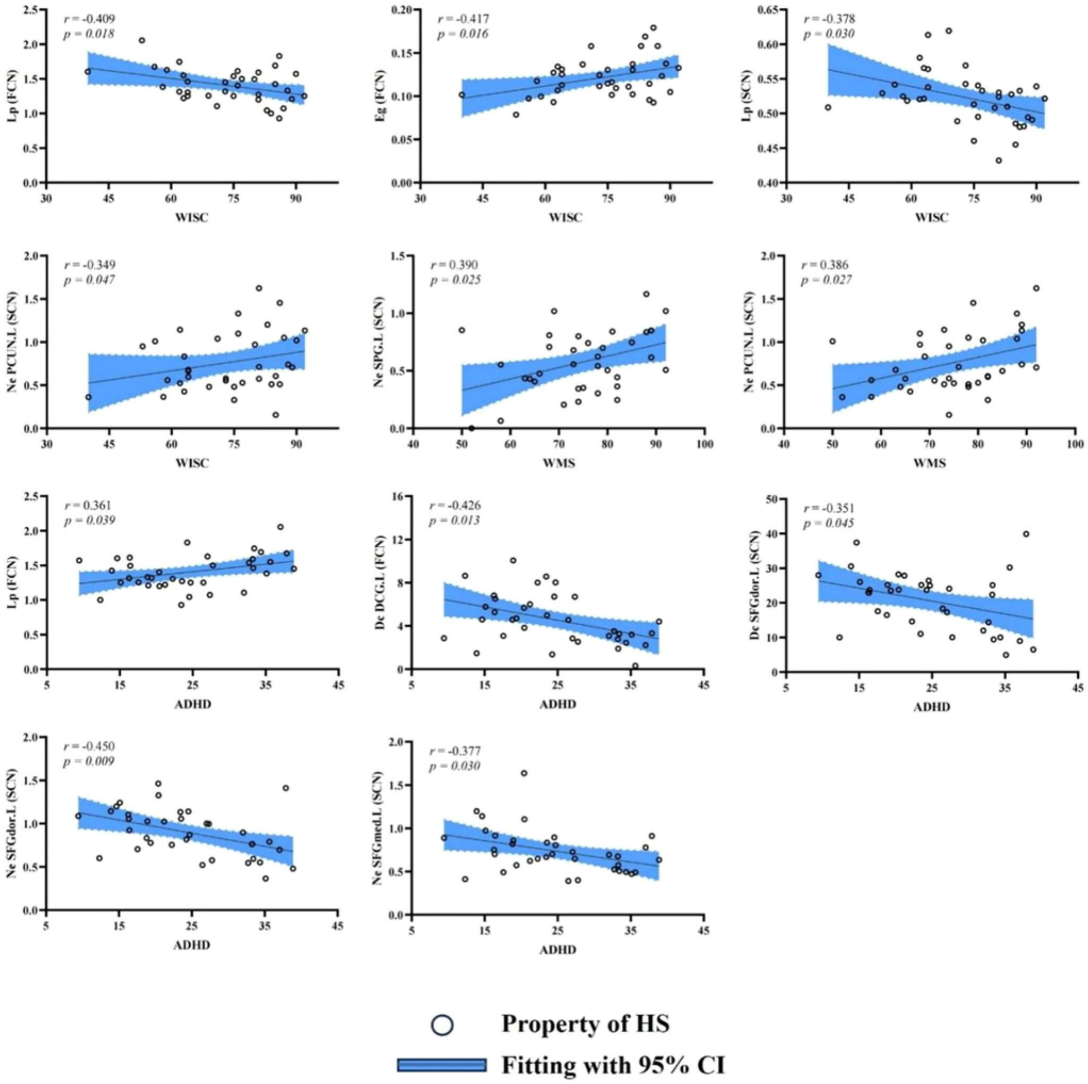
Partial correlation results between clinical scales and brain network properties. Partial correlation analyses were conducted to examine the relationships between the WISC, WMS, and ADHD scale scores in the HS group and brain network properties. *Note*: Age, sex, and disease duration were included as covariates to control for their effects on the results.

In the HS group, several statistically significant correlations were identified between clinical scale scores and brain network characteristics (Figure 5). For the WISC, both global properties, Lp of the FCN and SCN, showed negative correlations with WISC scores (*r* = −0.409, *p* = 0.018; *r* = ‐0.378, *p* = 0.030), while Eg of the FCN was positively correlated with WISC (*r* = 0.417, *p* = 0.016). In terms of node properties, the Ne of PCUN.L in the SCN was positively associated with WISC scores (*r* = 0.349, *p* = 0.047).

For the WMS, node properties indicated positive correlations between Ne of SPG.L and PCUN.L in the SCN and WMS scores (*r* = 0.390, *p* = 0.025; *r* = 0.386, *p* = 0.027). Lastly, concerning ADHD scores, global properties indicated a positive correlation between Lp of the FCN and ADHD (*r* = 0.361, *p* = 0.039). In the node properties, Dc of the DCG.L in the FCN as well as the SFGdor.L in the SCN showed negative correlations with ADHD (*r* = ‐0.426, *p* = 0.013; *r* = ‐0.351, *p* = 0.045). Additionally, Ne of the SFGdor.L and the SFGmed.L in the SCN demonstrated negative correlations with ADHD (*r* = ‐0.450, *p* = 0.009; *r* = ‐0.377, *p* = 0.030).

## 4 Discussion

HS is a rare condition stemming from hypothalamic damage, often due to tumors like craniopharyngiomas, which are the most common and can worsen HS post‐surgery in children's suprasellar region (Müller et al. 2022, van Santen et al. 2023). Typically classified as benign (World Health Organization [WHO] grade I), craniopharyngiomas often exacerbate HS after surgical resection (Jagannathan et al. 2005, Müller et al. 2019). Furthermore, the treatment of craniopharyngioma primarily involves complete surgical resection, which helps to minimize tumor‐related confounding effects on our neuroimaging data. This study utilized multimodal MRI to analyze differences in structure‐function coupling and large‐scale structural/functional network topology (global and nodal), as well as their correlations with cognitive developmental scales, in children with HS following craniopharyngioma resection. Three key findings were revealed: (1) Both structural and functional topological properties exhibited modifications, yet no evidence of structure‐function decoupling was observed; (2) Compared to HC, the HS group demonstrated impairments in both SCN and FCN, with structural network disruptions being particularly pronounced; (3) Cortical and subcortical hub reorganization occurred in HS children, involving regions such as the superior frontal gyrus, middle frontal gyrus, superior parietal gyrus, precuneus, and cingulate gyrus, indicating their critical role in the pathogenesis of HS‐associated cognitive developmental deficits. Collectively, these results elucidate the mechanisms underlying early neurocognitive developmental impairments in HS patients through a multimodal neuroimaging perspective, integrating both structural and functional network analyses.

To assess brain network alterations in children with HS, we analyzed global performance and node specialization. Both HC and HS groups exhibited small‐world properties, indicated by high clustering coefficients and short average path lengths, suggesting that overall information integration in HS children's brain networks is not abnormal (Wang et al. 2020, Bullmore and Sporns 2009). Additionally, our HS cohort demonstrated elevated Lp in the SCN, along with decreased Eg and Eloc. Here, Lp measures spatial separation, Eg quantifies the efficiency of information transfer within the brain network, and Eloc typically measures local connectivity and information transfer efficiency (Chen et al. 2022, Zheng et al. 2021). This combination suggests reduced efficiency in information transmission and interaction among regions within the SCN. Similar patterns were observed in the FCN, with increased Lp and decreased Eg, indicating compromised information transmission and interaction among FCN regions in HS patients. Together, these findings demonstrate a diminished capacity for functional integration within the local brain networks of children with HS.

Analysis of node characteristics in children with HS indicates statistically significant damage to various brain regions, particularly in the frontal, parietal, and occipital lobes. This aligns with prior morphological studies on HS patients with suprasellar tumors, especially those undergoing subfrontal approaches (Fuster 2019). Compared to the HC group, the attributes that were reduced in the HS group included Dc and Ne. Ne indicates the local connectivity and efficiency among nodes, while Dc measures the direct connections and mediation roles of nodes, which determine their importance within the network. The integration of these perspectives suggests that the overall integration and regional segregation within the brain networks of children with HS are indeed disrupted. Notably, alterations in the SCN and FCN of HS patients showed asynchronous differences, with more extensive damage in the SCN compared to the FCN. The SCN supports the FCN structurally, while the FCN can influence the SCN through plasticity mechanisms (Koubiyr et al. 2021).

Furthermore, we found no statistically significant differences in SC‐FC coupling between the HS and HC groups, indicating that the connection between the SCN and FCN in HS patients has not yet exhibited signs of decoupling or disconnection. This phenomenon may arise from the brain's ability to maintain normal function through compensatory mechanisms. For example, the brain might activate other unaffected areas or enhance specific functional connections to compensate for the impaired regions (Lohse et al. 2022).

Our observations indicate that the efficiency and importance of certain key brain regions may change, potentially leading to functional impairments associated with HS. The affected nodes can be categorized into four major large‐scale functional connectivity networks as proposed by Yeo et al: the fronto‐parietal network (FPN), the default mode network (DMN), the visual network, and the Limbic Network (Long et al. 2019). Areas such as the superior frontal gyrus, middle frontal gyrus, and superior parietal lobule are crucial components of the FPN, which is vital for executive functioning, cognitive flexibility, and attention control (Marek and Dosenbach 2018, Menon et al. 2023). Changes in the FPN have been noted in disorders like AADHD, Alzheimer's disease, and schizophrenia, suggesting that FPN impairment can adversely affect attention and executive functions, leading to cognitive decline (Samea et al. 2019, Dennis and Thompson 2014). Additionally, nodes such as the precuneus and superior occipital gyrus, integral to the visual network, process visual stimuli and are linked to the primary visual cortex. Research indicates that children may experience visual impairments following craniopharyngioma resection, likely due to damage to white matter fiber tracts caused by the tumor or surgical procedures (Elliott et al. 2011). Our findings support the idea that these disruptions can lead to persistent neurological deficits. The medial superior frontal gyrus and precuneus are also part of the DMN, which is involved in self‐awareness, emotional regulation, and social cognition (Menon 2023). Furthermore, nodes related to the limbic system, including the medial and paracingulate gyri, play essential roles in working memory, spatial regulation, and emotional expression (Rolls 2019). The reduction in Dc in the DCG.L indicates diminished neural associations with other brain regions, suggesting a reduced transmission of cognitive and emotional information. These alterations may contribute to understanding the cognitive deficits, attention impairments, and visual disturbances observed in children who develop HS following craniopharyngioma resection. However, further research is necessary to elucidate the intricate relationships among these brain networks.

Partial correlation analyses demonstrate significant associations between numerous affected attributes of the FCN and SCN with the scores of the WISC, WMS, and ADHD scale in the HS group. In terms of global attributes, an increase in Lp within the FCN is negatively correlated with WISC scores in the HS group, while it is positively correlated with ADHD scores. Conversely, a decrease in the Eg is positively associated with WISC scores. Similar patterns are observed within the SCN, where an increase in Lp is also negatively correlated with WISC scores in the HS group, suggesting that disruption in both SCN and FCN may adversely affect disease progression. Furthermore, in the FCN, a reduction in Dc in the DCG.L is negatively correlated with ADHD scores, whereas in the SCN, as there are reductions in Dc and Ne in the SFGdor.L, as well as in Ne in the left medial frontal gyrus, are also negatively correlated with ADHD scores. Notably, a decrease in Ne in the PCUN.L is positively correlated with both WISC and WMS scores in the HS group, while there is a decrease in Ne in the SPG.L is positively correlated with WMS scores, thus supporting our previous assertion regarding functional impairments resulting from changes in the fronto‐parietal, default mode, and limbic networks.

However, our study has several limitations. It is a single‐center investigation with a small sample size due to the rarity of the study population, highlighting the need for future research to increase sample size. Additionally, nodes like SFGmed.L, SPG.L, and PCUN.L are involved in multiple network activities, complicating the definition of functional connectivity networks. We plan to explore large‐scale functional connectivity alterations in HS using independent component analysis. Furthermore, due to the lack of specific relevant clinical scales, we could only conduct a brief assessment of the corresponding functions. Future studies should aim to develop more subjective scales to investigate the emotional, memory, and cognitive functioning of children with HS, thereby providing stronger validation for our hypotheses regarding their functional network impairments.

In summary, our findings demonstrate that children with HS following craniopharyngioma resection exhibit altered topological properties in both structural and functional connectivity networks. These neural alterations are associated with cognitive developmental impairments related to HS. Our results underscore the importance of analyzing large‐scale brain networks from multiple perspectives and further elucidate the mechanisms underlying cognitive developmental deficits in HS patients.

## Author Contributions

**Shuang Li**: writing – original draft, methodology, investigation, visualization, software, and resources. **Junya Ma**: data curation and supervision. **Zanyong Tong**: data curation, validation, and investigation. **Hongting Jiang**: data curation and investigation. **Lusheng Li**: writing – review and editing. **Yuting Zhang**: writing – review and editing.

## Peer Review

The peer review history for this article is available at https://publons.com/publon/10.1002/brb3.70730.

## Supporting information




**Supplementary Material**: brb370730‐sup‐0001‐SuppMat.docx

**Supplementary Material**: brb370730‐sup‐0002‐SuppMat.docx

## Data Availability

The data that support the findings of this study are available from the corresponding author upon reasonable request.
