# Supplementary material for: Alterations of Brain Structural and Functional Connectivity Networks and Its Correlations With Cognitive Function in Patients With Hypothalamic Syndrome Following Craniopharyngioma Resection
Source: Brain Behav. 2025 Aug 27;15(8):e70730. doi: 10.1002/brb3.70730 (PMC12382729; doi:10.1002/brb3.70730)
Supplement: Supplementary file 1 — Supplementary Material: brb370730‐sup‐0001‐SuppMat.docx [file BRB3-15-e70730-s002.docx]

The rs-fMRI data were collected using a gradient echo-planar imaging sequence with the following parameters: repetition time (TR) = 2000 ms, echo time (TE) = 30 ms, flip angle = 90°, field of view (FOV) = 240 × 240 mm², number of slices = 35, slice thickness = 4 mm, and a total of 240 time points were acquired. 3D T1WI image data were obtained using a three-dimensional gradient echo sequence with the following parameters: TR = 7.4 ms, TE = 3.1 ms, flip angle = 12°, FOV = 256 × 256 mm², voxel size = 1 × 1 × 1 mm³, and 156 slices. DTI data were acquired using a single-shot spin-echo planar imaging (EPI) sequence with the following parameters: TR = 7865 ms, TE = 84.2 ms, slice thickness = 2 mm, FOV = 224 × 224 mm², voxel size = 2.0 × 2.0 × 2.0 mm³. Diffusion gradients were applied in the b0 and 32 additional directions, resulting in a total of 33 directional images, with 64 slices scanned.
