# Supplementary material for: Alterations of Brain Structural and Functional Connectivity Networks and Its Correlations With Cognitive Function in Patients With Hypothalamic Syndrome Following Craniopharyngioma Resection
Source: Brain Behav. 2025 Aug 27;15(8):e70730. doi: 10.1002/brb3.70730 (PMC12382729; doi:10.1002/brb3.70730)
Supplement: Supplementary file 2 — Supplementary Material: brb370730‐sup‐0002‐SuppMat.docx [file BRB3-15-e70730-s001.docx]

| FCN | Functional connectivity networks |
| --- | --- |
| SCN | Structural connectivity networks |
| HS | Hypothalamic syndrome |
| HC | Healthy controls |
| WISC | Wechsler Intelligence Scale for Children |
| WMS | Wechsler Memory Scale |
| ADHD | Attention Deficit Hyperactivity Disorder |
| Lp | Characteristic path length |
| Eg | Decreased global efficiency |
| Eloc | Local efficiency |
| Dc | Degree centrality |
| Ne | Nodal efficiency |
| DCG.L | Median cingulate and paracingulate gyri |
| SC-FC | Structural-functional connectivity |
| MRI | Magnetic Resonance Imaging |
| rs-fMRI | resting-state functional Magnetic Resonance Imaging |
| DTI | Diffusion tensor imaging |
| 3D T1WI | 3D T1-weighted imaging |
| FA | Fractional anisotropy |
| FN | Fiber number |
| Cp | Clustering coefficient |
| γ | Normalized clustering coefficient |
| λ | Normalized characteristic path length |
| σ | Small-worldness |
| Bc | Betweenness centrality |
| Nle | Node local efficiency |
| NCp | Node clustering coefficient |
| AUC | Area under the curve |
| SFGdor.L | Superior frontal gyrus, dorsolateral |
| ORBsup.R | Superior frontal gyrus, orbital part |
| ORBmid.R | Middle frontal gyrus, orbital part |
| SFGmed.L | Superior frontal gyrus, medial |
| SOG.L | Superior occipital gyrus |
| SPG.L | Superior parietal gyrus |
| PCUN.L | Precuneus |
| FPN | Fronto-Parietal Network |
| DMN | Default Mode Network |
